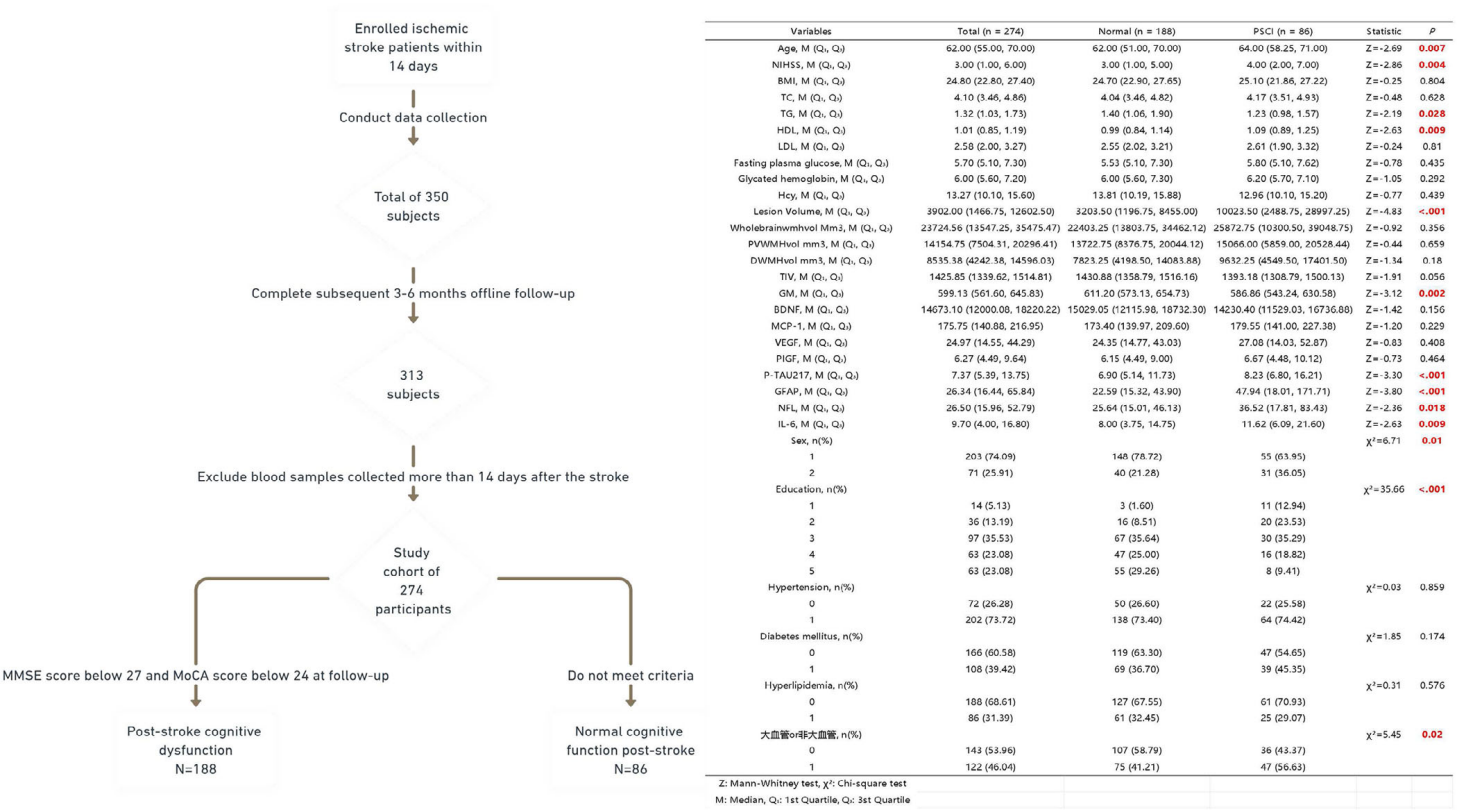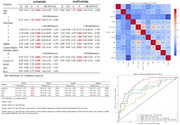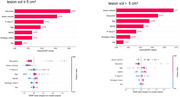# Predictive Value of pTau217 and Infarct Characteristics for Post‐Stroke Cognitive Impairment (PSCI): A Multicenter Cohort Study

**DOI:** 10.1002/alz70856_101954

**Published:** 2025-12-24

**Authors:** Mei Cui, Zishuo Jin, Yingzhe Wang, Qiang Dong, VICA investigators

**Affiliations:** ^1^ MOE Frontiers Center for Brain Science, Fudan University, Shanghai, China; ^2^ Huashan Hospital, Fudan University, Shanghai, Shanghai, China

## Abstract

**Background:**

The Global Burden of Disease Study 2019 reports that China has the highest number of stroke patients globally, with one‐third developing post‐stroke cognitive impairment (PSCI), making stroke‐related dementia the most prevalent form in China. Understanding the overlap between cerebrovascular pathology and Alzheimer's disease (AD) pathology is crucial in comprehending their joint contribution to cognitive decline post‐stroke.

**Method:**

This study, conducted within the Vascular, Imaging, and Cognition Association of China (VICA), included 350 patients enrolled within 14 days of ischemic infarction/TIA. Baseline blood samples were collected, and patients were followed up for six months for cognitive assessments. Of these, 274 patients completed the follow‐up and were divided into PSCI and PSNCI groups. Infarct lesions were quantitatively analyzed using diffusion‐weighted imaging (DWI), and several peripheral blood biomarkers were measured using a single‐molecule immunoarray, including, Aβ, pTau217, neurofilament light (NFL), glial fibrillary acidic protein (GFAP), monocyte chemoattractant protein‐1 (MCP‐1), matrix metalloproteinases (MMPs), interleukin‐6 (IL‐6), brain‐derived neurotrophic factor (BDNF), vascular endothelial growth factor (VEGF), and placental growth factor (PLGF).

**Result:**

Machine learning identified pTau217, GFAP, NFL, and IL‐6 most strongly associated with the occurrence of PSCI, which reflect neurodegeneration, inflammatory responses, and neuronal injury. After adjusting for factors such as infarct volume and NIHSS score, only pTau217 remained significantly associated with PSCI. In predicting the occurrence of PSCI, the area under the ROC curve (AUC) for pTau217 was 0.70, which was comparable to the AUC of 0.71 for infarct characteristics (including volume and location). However, the combination of pTau217 and infarct characteristics improved the prediction, achieving an AUC of 0.86. Notably, pTau217 demonstrated superior predictive performance for PSCI caused by smaller infarcts (lesion volume < 5 cm^3^). To further explore whether pTau217 reflects neurodegenerative pathology, we conducted amyloid PET imaging in a subset of patients with elevated pTau217 levels. The results confirmed that elevated pTau217 levels are associated with amyloid pathology.

**Conclusion:**

Pre‐stroke AD pathology may contribute to the development of PSCI. pTau217 serves as a valuable biomarker for detecting underlying amyloid pathology and holds promise in predicting PSCI.